# Some Points in the Treatment of Hemorrhoids

**Published:** 1881-08-20

**Authors:** William R. D. Blackwood

**Affiliations:** Pennsylvania


### SOME POINTS IN THE TREATMENT OF HEMORRHOIDS.

BY WILLIAM R. D. BLACKWOOD, M.D., OF PENNSYLVANIA.

In briefly considering this subject, I do not hesitate to assert at the outset that, aside from the actual suffering endured, no chronic malady causes more loss of time and money to its victims than the one under consideration; and when we remember the fact that many thousands are afflicted with hemorrhoids, the importance of the matter is readily apparent. It is unnecessary to refer to anatomical or pathological questions; these have already been thorougly discussed, and are understood by all practical surgeons. Piles are simply local anal tumors, varicose in nature, lying either or both within and without the sphincter, liable to inflame or ulcerate at intervals, caused by either local irritation or venous obstruction at points more or less remote, disorders of the hepathic system of vessels being notably productive factors. A "fit of piles," as the laity term the acute inflammatory action which sets in at intervals, is accompanied by general febrile disturbance, together with prostatic, vesical, and gastric irritability in the male, and in the female the bladder symptoms are frequently exchanged for serious uterine complications. Pregnancy, which frequently produces hemorrhoids, may be terminated through abortion, induced by acute inflammation of piles, especially in the case of those long affected.

The principal agents productive of hemorrhoids are errors in the digestive function, through inattention to diet, neglect to secure a full, free and daily alvine evacuation, or the production of harsh cathartic action in constipated persons through aloetic purgatives especially; the vaunted use of aloes in the treatment of piles, as lately advocated, to the contrary notwithstanding. Onanism, and certain method of generaic fraud, especially tend to the production of hemorrhoids in both sexes, and aggravate the condition existing. Every successive "fit" increases the trouble already present, and ulceration is intensified, thus increasing the liability to hemorrhage. I am not a believer in pathological safety-valves or drains, and unless in the case of typical gormandizers, who must bleed or burst—and for them this is as convenient an outlet as any other—I always interfere, when hemorrhage becomes free, or repeated at short intervals; and I may here state that I have operated under very diverse conditions, both for hemorrhoids and fistula, and have never seen anything but good result, in spite of the popular notion respecting the danger of so doing during the coexistence of pulmonary and other complaints.

The first point in the medical management of a case is strict attention to diet. We extend our gastronomic performances too much in this country, and the national virtue of getting away with our meals at breakneck speed is proverbial, especially in our traveling public, whom necessity compels to eat too often bad food villainously cooked, eating-house bicuits particularly, being beyond even the power of an ostrich to digest, as no doubt many of my hearers know from personal experience. It has been said by some Solon that "every man should be his own doctor at forty;" and, in my opinion, all men should, if they deserve to live at all, know what diet suits them at half that age, and adhere to it. As few persons, however, attend to this matter, it behooves the physician to carefully watch his hemorrhoidal patients in this respect. Fruit should enter largely into the dietary, and I have found excellent results follow the habitual, daily use of at least a pint of the juice of the ordinary tomato, and it should preferably be uncooked. This esculent can be readily preserved throughout the year in any of the numerous air-tight cans or jars in common use. In lieu of baker's or home-made white wheat bread, Graham, oaten or bran bread and crackers should be taken, and a bowl of gruel made from either oat or Indian meal is valuable at bedtime in constipated habits.

Instead of common salt, the addition of a few grains of sodium phosphate acts happily. All alcoholic, malt, or other liquors, from the strongest to the mildest, including home-made beverages, must be strenuously tabooed, and many sufferers are greatly relieved simply through abstinence in this direction, venous congestion of the hepatic system being often unconsciously maintained through moderate indulgence in drinking. Regularity in eating is essential; better miss the meal than partake an hour too soon or too late. Next to diet, but not less important, is the necessity of a full, free and daily evacuation of the colon and rectum, preferably before commencing the duties of the day. As in everything else, habit has much to do with this, and the bowel can, in the majority of cases, be educated to unload itself without medication. On rising from bed or breakfast, gentle massage of the abdomen, having first bathed it rapidly with fresh cold water, and dried by thorough friction with a rough towel, will in a short time so tone the muscles, of the parietes and the bowel as to compel action, even in obstinate complication. I have repeatedly relieved this miserable condition by this simple process without a solitary dose of any medicine. The application of induction current from a good Faradic battery replaces massage, but must be kept in the hands of the physician, as injudicious and too powerful currents over the solar plexus of the sympathetic will frequently induce faintness and depression. If medication must be used, or obstinate hepatic torpidity persists, an admirable combination is one minim (.066 c. c.) each of ext. fl. euonymin, iridin, and tr. belladonna, with or without strychnia. This may at times be replaced by similar small doses of Fowler's solution, fl. ext. phytolacca, and tr. belladonna, and whichever is used should be repeated four times daily. Fluid preparations are preferable because of their reliability and facility of absorption. To obtain good results, the administration must be maintained for several weeks, or until the case is evidently in need of surgical interference. The use of ergot and glycerine internally has been negative with me. An enema of lukewarm water before defecation is valuable in ulcerated cases, and water mopped on as hot as bearable after a motion will relieve the hemorrhage, if severe. *Paper of any kind should never be used in the closet*, but in place thereof, a soft sponge, with carbolated water, should be freely applied to cleanse the mass before replacing it inside the sphincter. *This point is exceedingly important* and should be insisted on by the attendant. All supporters, ointments, suppositories, and the like local applications have utterly failed in my practice, and I have tried many highly lauded.

A faithful trial of such medical treatment failing, operative measures should at once be instituted. My plan is, to urge operation after the first well-defined "fit," or acute inflammatory attack, for no one knows how soon the next, and possibly severe one, may ensue. I also snip off all external piles, or shrunken tabs, as soon as discovered, to prevent transfer of the irritation to which they are peculiarly exposed to co existing internal hemorrhoids. For many years my operative measures were confined to the ligature, nitric acid, and the galvano-cautery, but for the last three years I have used exclusively, in all cases, pure crystalized carbolic acid, enough glycerine only being added to insure fluidity. With this the masses are injected—two at a time if small, one only if large. In very large tumors the acid is deposited in two or more points without entirely withdrawing the needle, and the body of the pile is injected alone, it being insensitive, whilst dilute solutions are absorbed more or less, as evidenced by the taste in the patient's mouth. The stronger acid is therefore the better, as avoiding probable depression through absorption. Morphia may be added or used subsequently to the injection; anæsthesia is not necessary. The injection should be made slowly, complete rest enjoined, solubility of the bowel insured, light diet allowed, and the cure is assured, without danger, which is more than can be said of any other method.

During the last twenty years my experience has been large, and my deductions are based entirely upon practical results, not upon theory. I am convinced that the subject does not receive the attention it deserves, and with a desire to attract more attention to it, and to summarize in closing, the following points are suggested :

1. Hemorrhoids may be arrested by proper attention to diet, and to a normal daily evacuation of the bowel.

2. Hemorrhoids, being present, may be generally kept in check, frequently greatly relieved, and sometimes cured entirely, by the means used to prevent them.

3. Hemorrhoids becoming troublesome, despite medical treatment, should be removed surgically without delay.

4. Hemorrhoids may be quickly, surely, and safely removed by the preferable operation of injection by carbolic acid.

